# Erratic Asian summer monsoon 2020: COVID-19 lockdown initiatives possible cause for these episodes?

**DOI:** 10.1007/s00382-021-06042-x

**Published:** 2022-01-24

**Authors:** Ramesh Kripalani, Kyung-Ja Ha, Chang-Hoi Ho, Jai-Ho Oh, B. Preethi, Milind Mujumdar, Amita Prabhu

**Affiliations:** 1 Indian Institute of Tropical Meteorology, Pune, 411008 India; 2 Center for Climate Physics, Institute for Basic Science, Busan, South Korea; 3 Research Center for Climate Sciences and Department of Atmospheric Sciences, Pusan National University, Busan, South Korea; 4 School of Earth and Atmospheric Sciences, Seoul National University, Seoul, South Korea; 5 Nano C&W, Gyeonggi-do, South Korea; 6 Center for Climate Change Research, Indian Institute of Tropical Meteorology, Pune, India; 7 Radar and Satellite Meteorology Project, Indian Institute of Tropical Meteorology, Pune, India; 8 Residence: B-303 Sai Royale Society, Wanowari, Pune 411040 India

**Keywords:** Asian summer monsoon, COVID-19, Lockdown, Aerosol, Climate change, Extremes

## Abstract

The summer (June through September) monsoon 2020 has been very erratic with episodes of heavy and devastating rains, landslides and catastrophic winds over South Asia (India, Pakistan, Nepal, Bangladesh), East Asia (China, Korea, and Japan), and Southeast Asia (Singapore, Thailand, Vietnam, Laos, Cambodia, Philippines, Indonesia). The withdrawal of the summer monsoon over India was delayed by 2 weeks. The monsoon season over East Asia has been the longest. China recorded a Dam burst in the twentieth century. Furthermore, the Korean Peninsula has experienced back-to-back severe tropical cyclones. Could the lockdown activities initiate to control the COVID-19 spread a possible cause for these major episodes? The strict enforcement of the lockdown regulations has led to a considerable reduction of air pollutants—dust and aerosols throughout the world. A recent study based on satellites and merged products has documented a statistically significant mean reduction of about 20, 8, and 50% in nitrogen dioxide, Aerosol Optical Depth (AOD) and PM_2.5_ concentrations, respectively over the megacities across the globe. Our analysis reveals a considerable reduction of about 20% in AOD over South as well as over East Asia, more-over East Asia than over South Asia. The reduced aerosols have impacted the strength of the incoming solar radiation as evidenced by enhanced warming, more-over the land than the oceans. The differential warming over the land and the ocean has resulted in the amplification of the meridional ocean-land thermal contrast and strengthening of the monsoon flow. These intense features have supported the surplus transport of moisture from the oceans towards the main lands. Some similarity between the anomalous rainfall pattern and the anomalous AOD pattern is discernable. In particular, the enhancement of rainfall, the reduction in AOD and the surface temperature warming match very well over two regions one over West-Central India and the other over the Yangzte River Valley. Results further reveal that the heavy rains over the Yangzte River Valley could be associated with the preceding reduced aerosols, while the heavy rains over West-Central India could be associated with reduced aerosols and also due to the surface temperature warming.

## Introduction

The Asian summer monsoon, the most robust global monsoon system, can be broadly classified into two main subsystems the South Asian summer monsoon (in particular the Indian summer monsoon) and the East Asian summer monsoon (comprising monsoons over China, Korea and Japan) (Ha et al. 2017 and references therein). The South Asian Summer monsoon, which lasts for 4 months from June through September, contributes about 80% of the annual rainfall, whereas the East Asian summer monsoon contributes about 60% of the annual rainfall during the June through August period (Ha et al. 2012). These monsoons impact the livelihood of nearly half of the world’s population and play a significant role in the large-scale climate variability over much of the globe. Although the South and East Asian regions are geographically distant and independent of each other with different life styles, their monsoon systems interact with each other on different time scales. The known connections between these two subsystems have been a subject of research for at least 6 decades (e.g. Flohn 1958). Multiple studies have carried this work into the twenty-first century and have examined the in-phase and out-of-phase inter-annual variations between the South and East Asian Monsoons. (e.g. Kripalani and Kulkarni 2001; Zhang 2001; Wu 2002, 2017; Greatbatch et al. 2013; Vaid and Liang 2015; Ha et al. 2017; Preethi et al. 2017; Prabhu et al. 2018; Yong and Huang 2019; Vaid 2019; Kim et al. 2020 and many others). The connection of the summer rainfall variation over the two monsoons have been linked through two pathways (Wu 2017). One is through the atmospheric circulation over the lower latitudes that involves the North Pacific Sub-Tropical High (NPSH) and the East Asia–Pacific Japan tele-connection pattern and the other one is via an extratropical Silk Road/Circum-global Tele-connection (CGT) pattern along the upper-level westerly jet stream (Ding and Wang 2005; Kripalani et al. 1997; Lee and Ha 2015).

It has been well documented that the extensive Asian Summer monsoon system is essentially driven by the large-scale thermal contrast arising from the different heat capacities of land and ocean in response to the seasonal changes in solar radiation reaching the Earth’s surface. An alternative hypothesis in which the basic system responsible for the monsoon is considered to be the Inter-tropical Convergence Zone (ITCZ) or the elongated trough moving northwards from the equatorial regions towards the Indian mainland has been proposed (Gadgil 2018). The South Asian monsoon is dominated by the elongated low-pressure zone, the quasi-stationary monsoon trough over the Indo-Gangetic Plains and the East Asian monsoon is dominated by the frontal zone called as Meiyu in China, Changma in Korea and Baiu in Japan. Besides the dominant roles of the ITCZ and the ocean-land thermal contrast, aerosols from both natural and anthropogenic emissions can further modulate the monsoon system. The heavy aerosol layers can cool the surface by scattering and absorbing solar radiation, which reduces the ocean-land thermal contrast and in turn, weakens the monsoon system the so-called solar dimming or global warming (Jin et al. 2021).

Using a comprehensive set of high spatio-temporal resolution satellites merged products of air pollutants, Manmeet et al. (2021) analyzed the air quality across the globe and quantified the improvement resulting from the suppressed anthropogenic activity during the lockdowns initiated to control COVID-19 spread. A statistically significant mean reduction of 19.7, 7.4, and 50% in nitrogen dioxide, Aerosol Optical Depth (AOD) and PM_2.5_ concentrations, respectively over the megacities across the globe has been. Several other recent studies have also documented considerable reduction in AOD levels (e.g. Kumar 2020; Singh and Chauhan 2020; Kumari and Toshniwal 2020; Bhawar et al. 2021) Model-based analysis also documents reduced aerosol amount (particularly over southern and eastern Asia) and associated increase in surface shortwave radiation levels due to COVID-19 regulations (e.g. Jones et al. 2021; Fadnavis et al. 2021). Thus, recent studies on the variation of air quality have documented a considerable reduction in air pollutants/aerosols over South as well as East Asia. These reduced aerosols could possibly impact the incoming solar radiation, enhance the land–ocean thermal contrast, strengthen the atmospheric circulation and eventually modulate the rainfall distribution.

Highly unusual and unprecedented heavy amounts of rainfall were experienced during the summer monsoon 2020 over India. India had one of its wettest monsoons since 1994. The month of August 2020 was the wettest on record over India and Pakistan. Heavy rains, flooding and landslides also affected the surrounding countries. Persistent high rainfall in the Yangzte River basin in China caused severe flooding. China also recorded the longest rainy season and highest precipitation since 1961. Korea experienced its third wettest summer and longest rainy season since 1973. Parts of western Japan were also affected by significant flooding. The highest precipitation since 1946 was recorded in Japan’s Kumamoto province (APCC 2021; WMO 2021). In view of above, it worth investigating: Were the unprecedented heavy rains over South Asia and East Asia during monsoon 2020, a part of natural variability, or the effect of global warming or the impact of the possible re-distribution of aerosols and environmental changes due to lockdown initiatives to control the spread of COVID-19 pandemic?

To seek the possible answers for the above questions, observational data has been used. The structure of this study is laid out as follows: The data and the methodology are described in Sect. 2. Climatic features such as the lower tropospheric monsoon circulation, sea surface temperatures over the Indian and the Pacific Oceans, moisture flux transports and convergence are examined in Sect. 3 to ascertain the possible climatic features/causes for these unprecedented heavy rains. The possible impact of the COVID-19 lockdown initiatives on aerosol distribution and air quality is discussed in Sect. 4. Section 5 describes the role of aerosols on monsoons, and Sect. 6 examines the possible impact of the aerosol distribution changes due to lockdown initiatives on the monsoon 2020 heavy rains. Finally, discussion and summary are detailed in Sect. 7.

## Data and methodology

(i) The study utilizes the monthly precipitation and mean 2 m temperature, at a horizontal resolution of 0.5° × 0.5°, obtained from Climate Research Unit time series (CRU TS; Harris et al. 2020).
(ii) In addition, the daily precipitation from the Global Precipitation Climatology Project (GPCP) having a horizontal resolution of 2.5° × 2.5^o^ (Adler et al. 2017) has been used.
(iii) Circulation datasets at a resolution of 2.5° × 2.5° are taken from the National Center for Environmental Prediction/National Center for Atmospheric Research (NCEP/NCAR) reanalysis products (Kalnay et al. 1996).
(iv) Sea Surface Temperatures (HadISST; Rayner et al. 2003) having a resolution of 1° × 1° developed at Met Office Hadley Centre for Climate Research have also been used.
(v) Apart from these datasets, the study also uses Aerosol Optical Depth (AOD) analysis, with a horizontal resolution of 0.625° × 0.5°, from the second Modern-Era Retrospective Analysis for Research and Applications (MERRA-2) reanalysis product (GMAO 2015; Randles et al. 2017).

Simple techniques of compositing, differencing and anomaly correlation coefficients are employed to get the desired results. For computing the anomaly correlation coefficients, we have directly used the NCAR Command Language (NCL) script available at (https://www.ncl.ucar.edu/Document/Functions/Contributed/pattern_cor.shtml).

## Distinct climatic characteristics of monsoon 2020

All mean patterns are based on the 20 years period (1996–2015). Climatological mean rainfall features over the Asian land-mass reveal large amounts of rainfall located over the west coast of India, central and northeast India, Arakan/Myanmar coast, region of Southeast Asia extended till the Philippines and southeast China till the Korea-Japan peninsula (Fig. 1a). This mean pattern is identical to the pattern based on data for 44 years (Preethi et al. 2017). A similar spatial rainfall pattern prevails during the summer monsoon 2020 (Fig. 1b) but with much higher rain intensities. However, the difference between the above two panels (Fig. 1b, a) depicts the anomalous excess rainfall exceeding 1–2 mm/day over peninsular India including the West Coast of India (Fig. 1c) and over a broad band region extending from northeast India through to the Yangzte River Basin in China up to the Korea-Japan peninsula (Fig. 1c). Such a broad band with in-phase rainfall anomalies starting from northeast India through China to the Korean peninsula have been suggested in an earlier study (Kripalani et al. 1995). Incidentally, several regions over Southeast Asia including Myanmar and the regions surrounding the South China Sea – the northern Philippines to the east and Vietnam/Laos on the west side of the South China Sea display deficit rainfall of nearly 1–2 mm/day.

**Fig. 1:**
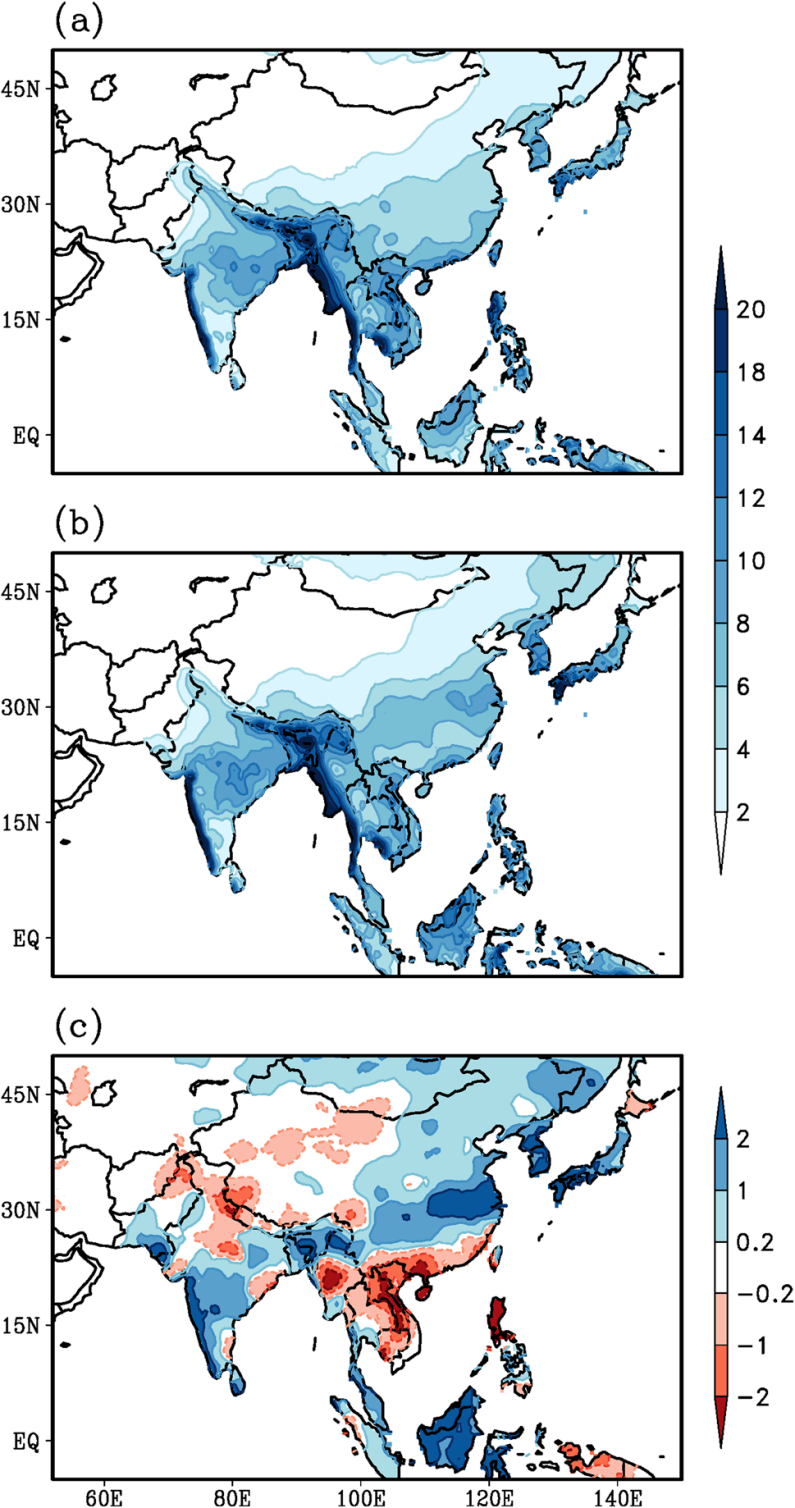
Spatial variation of summer monsoon rainfall (mm/day) as derived from CRU rainfall dataset. **a** Climatology for the period 1996–2015, **b** monsoon 2020 and **c** anomaly for the monsoon 2020, obtained by subtracting (**a**) from (**b**)

India received 109% of Long Period Average (LPA) rainfall during the 2020 summer monsoon, making the wettest on record after 1994 (112% of LPA) and 2019 (110% of LPA). Cyclones Amphan and Nisarga are playing a role in dragging the equatorial ITCZ onto the Indian landmass to form the monsoon trough and avoiding a potential delay in the onset. August was the wettest during the last 44 years. Mumbai and Goa along the west coast of India experienced the wettest monsoon in over 60 years (India Meteorological Department 2020a; Murtugudde 2020). Above normal rainfall occurred over much of peninsular India (Fig. 1c). During August, the formation of back-to-back low-pressure systems over the northern Bay of Bengal and their movement towards west and northwest caused higher than normal rainfall over central and western India. August recorded 127% of average August rainfall, the fourth highest in 120 years (India Meteorological Department 2020b; Murtugudde 2020). High August rainfall is clearly supported by the south to north rainfall propagation from the equatorial regions up to 25°N over the Indian longitudes (Fig. 2a) commencing from mid-July till the beginning of September. September also recorded rainfall on the positive side—again supported by another northward propagation from beginning till the end of September (Fig. 2a). The weak monsoon in July was mainly due to the absence of major monsoon disturbances over the Bay of Bengal. This also led the monsoon trough close to the foothills of the Himalayas leading to heavy rains over the foothills of the Himalayas, in particular over the northeast Indian region. Alternate wet and dry spells prevail during July between 10 and 25^0^ N (Fig. 2a). The withdrawal of the monsoon was delayed by about 2 weeks. A recent study (Vaid and Kripalani 2021b) witnessed a dramatic variation and interaction of the upper tropospheric temperature with one centered over the Tibetan Plateau and the other over the west Pacific resulting in heavy monsoon rains during summer 2020 over the Indian sub-continent.

**Fig. 2:**
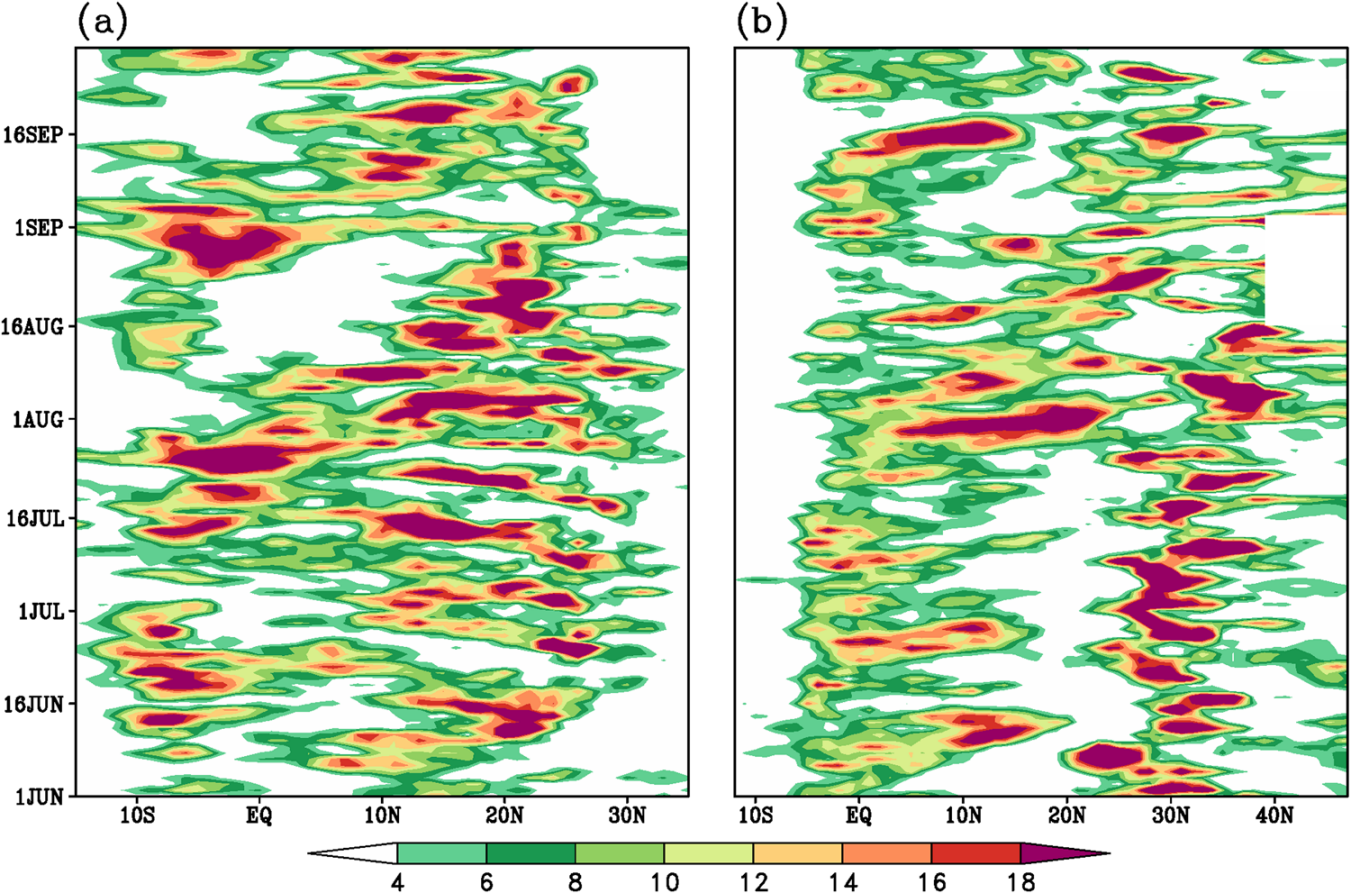
South to north propagation (time-latitude section) of rainfall (mm/day) averaged over the **a** South Asian (70°E–90°E) and the **b** East Asian (110°E–140°E) longitude belt, during the summer monsoon season of 2020. GPCP daily rainfall data is used to derive the rainfall propagation characteristics

Over East Asia, during summer 2020 Yangzte River Basin (25–35°N, 105–120°E) experienced persistent Meiyu rainfall and suffered from severe/ catastrophic flooding***.*** The accumulated rainfall exceeded that in 1998 and broke its record since 1954 with frequent heavy rainfall events occurring mainly in June and July (Liu and Ding 2020; Liu et al. 2020; Clark et al. 2021; Ding et al. 2021). South Korea also experienced one of its most unusual East Asian Summer Monsoon seasons since 1973 due to the very active Changma front. Torrential rains wreaked havoc across the country causing landslides, flooding and taking lives (Park et al. 2021). In July, ongoing heavy rains affected eastern and western areas of Japan in association with the stagnation of an active Baiu front over mainland Japan. Monthly precipitation on the Sea of Japan side of eastern Japan and in western Japan were the highest recorded. Precipitation amounts during June–August were significantly above normal. The rainy season ended later than usual over South Korea as well as over Japan (TCC News 2021). The continuous heavy rainfall spells over Yangzte River Valley – South Korea- Japan are clearly visible with continuous rain bands—as evidenced over the East Asian longitudes (110–140°E) between 25 and 35°N commencing in June up to mid-August (Fig. 2b).

### Lower tropospheric circulation

The dominant southwesterly flow at 850 hPa over the Indian sub-continent (Fig. 3a) is evident from the southern Indian Ocean through the Arabian Sea penetrating the Indian landmass and entering the Bay of Bengal where it curves to a southeasterly flow over the head Bay of Bengal to form the monsoon trough over the Indo-Gangetic plains. Over the East Asian sector, the NPSH with its western edge along 120°E is the dominant system transporting moisture from the west Pacific towards south China, South Korea and Japan. A stronger monsoon flow prevails during monsoon 2020 with the southwesterly flow from the Bay of Bengal penetrating even into the China landmass (Fig. 3b).

**Fig. 3:**
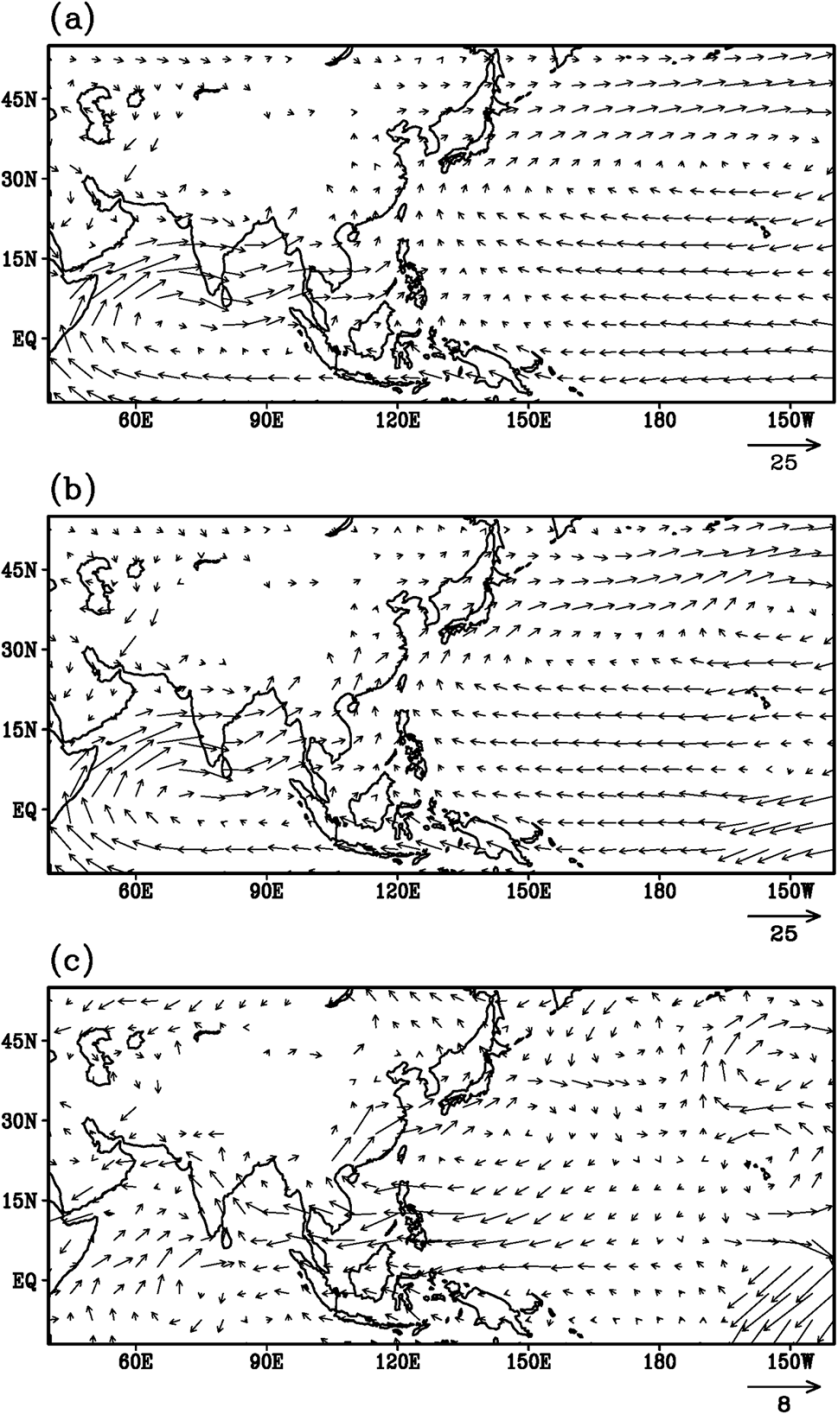
Lower tropospheric (850 hPa) circulation (m/s) during summer monsoon season, derived using NCEP-NCAR reanalysis product. **a** Climatology for the period 1996–2015, **b** monsoon 2020 and **c** anomaly for monsoon 2020

The anomalous wind pattern (Fig. 3c) reveals strengthening of the southwesterly flow over the Arabian sea, in particular the southerly component appears much stronger than its westerly component over the eastern Arabian Sea. Enhancement of the south-westerly flow over Arabian (5°S to 15°N, 55°E to 80°E) is clearly visible (Fig. 3c) as well as over East Asia along 105°E north of 10°N. Over the East Asian sector, the western edge of the NPSH has shifted to about 110°E. Such large westwards shifts of the NPSH have been observed during recent decades (Mujundar et al. 2012; Preethi et al. 2017). In summer 2020 an anomalous westward extension of the NPSH was detected (Liu and Ding 2020), transporting more moisture to the Yangste basin from the tropical Ocean and enhancing the Meiyu rainfall amount (Li and Lu 2018). This exceptional event of the displacement of the NPSH has been attributed to the Indian Ocean SST and mid-latitude wave train emanating from North Atlantic (Liu et al. 2020). Furthermore, the easterlies at the southern edge of the NPSH have penetrated into the Bay of Bengal and over central parts of India. Interestingly, the penetrating easterlies of the NPSH over the India sub-continent along with the southwesterly over the Arabian Sea converge over the west coast of India, resulting into an anomalous cyclonic circulation centered at 15°N, 70°E (Fig. 3c). This anomalous cyclonic circulation may have transported moisture supply from the Arabian sea resulting in heavy rainfall amounts over peninsular India and the West-Coast of India (Figs. 1c, 3c).

The Korean peninsula was wedged between very persistent anomalous high pressure, one located south of Japan and a smaller and weaker one over northeast of Japan (Fig. 3c). Whereas the southern system the NPSH provided the moisture for Korean Changma, the northern one created northerly (southward) winds which held the moisture in place over the Korean peninsula creating more persistent rains (Oh et al. 2018).

### Sea surface temperatures

The SST mean pattern (Fig. 4a) and the pattern during monsoon 2020 (Fig. 4b) are similar with warmer waters over the West Pacific Ocean and the eastern Indian Ocean. However, the difference (Fig. 4b, a) clearly reveals a basin-wide warming over the Indian Ocean and the development of the La Nina phase of the El Nino Southern Oscillation (ENSO) phenomenon over the Pacific—with positive SST anomalies over the West Pacific and negative SST anomalies over the equatorial central and eastern Pacific (Fig. 4c). In summer 2020 the SSTs around the South China Sea, the Maritime continent and the north Pacific adjacent to the American coast were also warmer than normal.

**Fig. 4:**
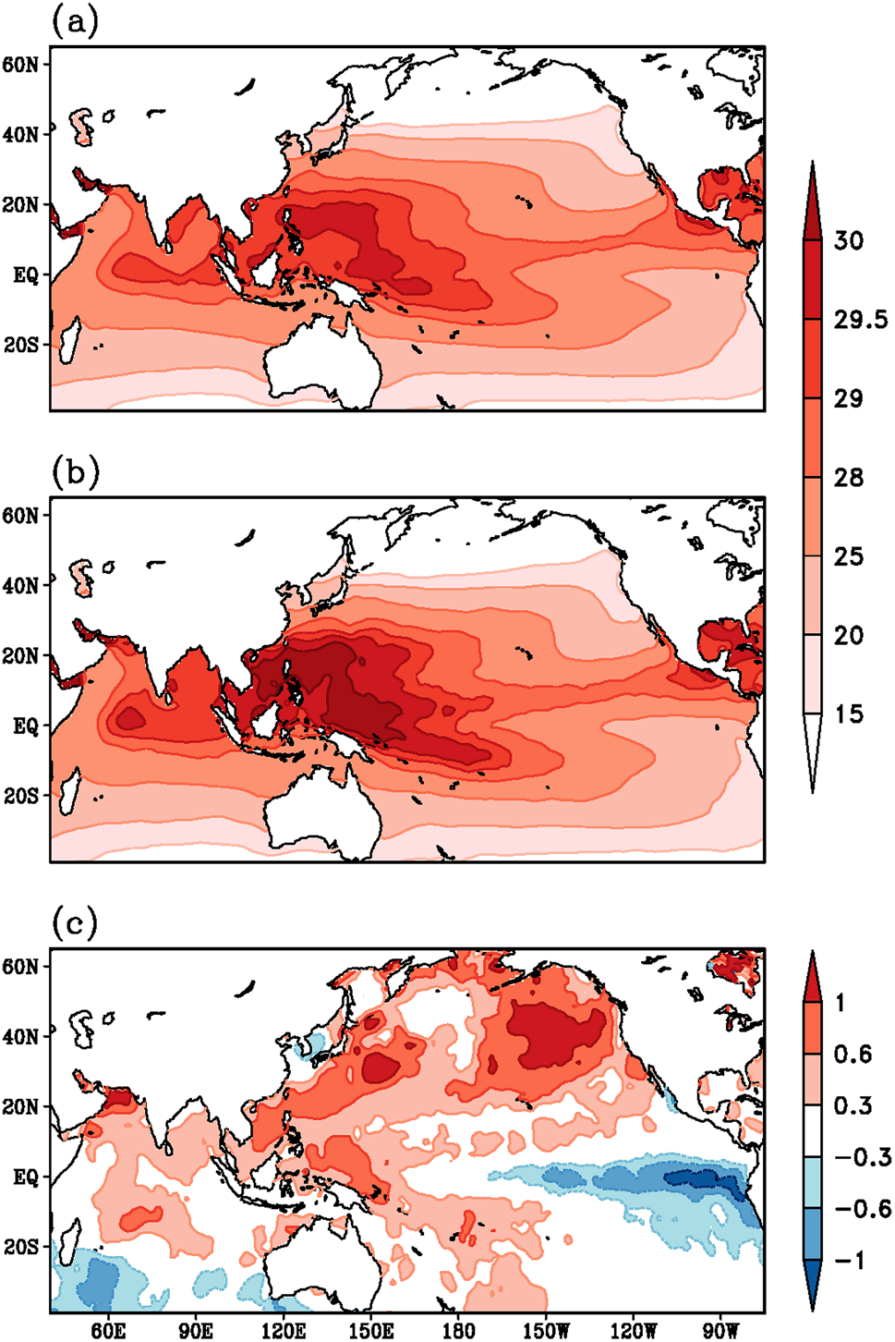
Spatial variation of sea surface temperature (°C) during summer monsoon season, as derived from HadISST datasets. **a** Climatology for the period 1996–2015, **b** monsoon 2020 and **c** anomaly for monsoon 2020, obtained by subtracting (**a**) from (**b**)

Recent studies point out that the basin-wide Indian Ocean warming in summer 2020 could have been induced by the exceptional persistent simultaneous Madden Julian Oscillation (MJO) activity (Zhang et al. 2021b) and the extreme positive Indian Ocean Dipole (IOD) in 2019 (Takaya et al. 2020; Zhang et. al. 2021a) which contributed to the NPSH and the enhanced the Meiyu rainfall (Zhou et al. 2021). Such a delayed impact of the IOD on the subsequent summer monsoon rainfall over East Asia- West Pacific has been reported earlier (Kripalani et al. 2010). Wang (2020) attributed the long-lasting Meiyu season during summer 2020 to the co-existence of the Silk-Road wave train in the upper troposphere and the Pacific-Japan wave-train in the middle-lower troposphere. This has favored the movement of the South Asian High and the NPSH towards each other leading to heavy rains. Westward movement of the NPSH has been noted in Sect. 3.1

Ha et al. (2017) suggested that there are two integral views of the inter-connection between these two monsoon systems, one is that the positive correlation which is associated with the decaying El Nino and developing Indian Ocean SST warming anomalies, the other is the negative inter-connection resulting from developing El Nino and the western Pacific SST cooling (Ha et al. 2017). Incidentally, the winter state of 2019 was a mild El Nino and it transitioned into a La Nina state during the summer of 2020, as evidenced in Fig. 4c. Besides, the India Ocean SSTs also display positive SST anomalies (Fig. 4c). Thus, the decaying El Nino and the warming of the Indian Ocean may be one of the factors leading to the positive in-phase above normal rainfall variations over both the monsoon systems during the summer of 2020 (Fig. 1c).

### Moisture flux transport and convergence

Various pathways transporting moisture from the South Asian region towards the East Asian region and vice-versa have been proposed (e.g. Zhang 2001; Huang et al. 2017; Yong and Huang 2019) between the in-phase as well as out of phase relationships between these two monsoon subsystems. The vertically integrated (from surface to 300 hPa) moisture transport and convergence mean pattern (Fig. 5a) and the pattern for the summer monsoon 2020 (Fig. 5b) reveal similar features, however with more intense features during the monsoon 2020 (Fig. 5b). The westward shift of the large-scale circulation tends to weaken the moisture convergence, induced by easterly trades and westerly mean monsoon winds, over south-west Pacific (100–135°E, 10–30°N) during 2020 (Fig. 5b and c) relative to the climatology (Fig. 5a), resulting in deficit rainfall activity over southeast Asia and the regions surrounding the South China Sea. Also, the anomalous moisture outflow over the north-central Indian sub-continent seems to be modulated by deep penetration of anticyclonic easterly flow over the west Pacific and Bay of Bengal (Fig. 5c). Interestingly, this anomalous moisture flows across the Indian sub-continent in turn produces anomalous cyclonic moisture convergence across the entire West-Coast of India and adjacent Arabian Sea as seen in Fig. 3c. Such anomalous moisture transport features across the Indo-Pacific sector during 2020 are consistently supported by the variability of tropical convection and upper-level circulation feature described in earlier studies (Mujumdar et al. 2012; Priya et al. 2015; Preethi et al. 2017). Furthermore, the southwesterly flow over the Bay of Bengal and the southerly flow at the western edge of the NPSH appear to converge and meet around 20°N, 105°E (Fig. 5b), suggesting the possibility of the moisture from the Bay of Bengal as well as from west Pacific—South China Sea converging over southern parts of China—South Korea -Japan, resulting in the unprecedented torrential heavy rains over the Yangzte River Valley and the Korean peninsula. Finally, the westward shift of the NPSH is also conducive for transporting moisture from the South China Sea towards the Indian subcontinent as evidenced by several westward propagations of rainfall bands from about 130^0^E to 70^0^E throughout the monsoon season (Fig. 6). Such westward transports of moisture from the South China Sea towards the Indian subcontinent have been noted in a recent study (e.g. Vaid and Kripalani 2021a).

**Fig. 5:**
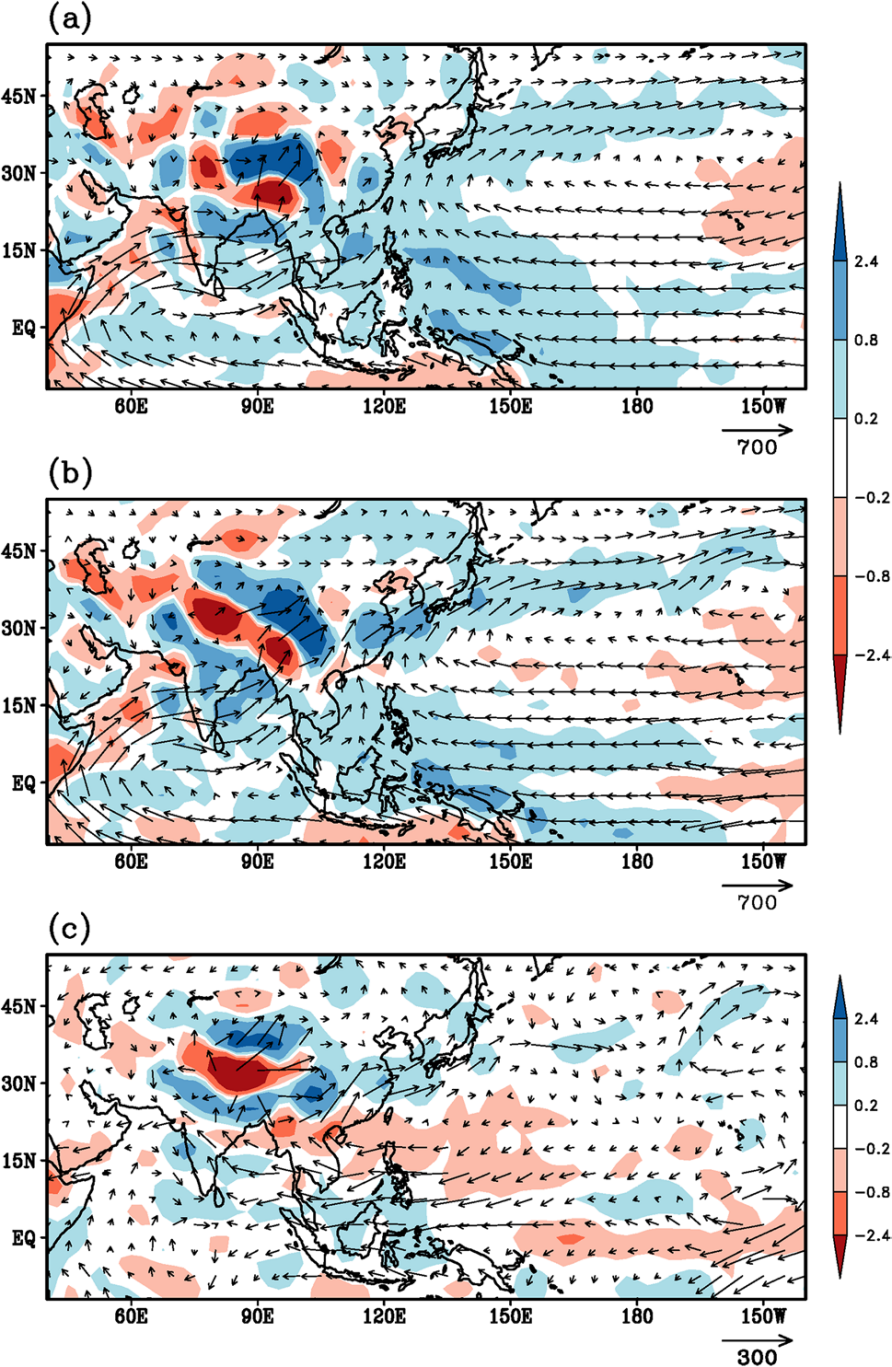
Vertically (from surface to 300 hPa) integrated moisture transport (Kg m^−1^ s^−1^ vectors) and moisture convergence (× 10^–4^ kg m^−2^ s^−1^, shadings) during summer monsoon season, derived using NCEP-NCAR reanalysis product. **a** Climatology for the period 1996–2015, **b** monsoon 2020 and **c** anomaly for monsoon 2020

**Fig. 6:**
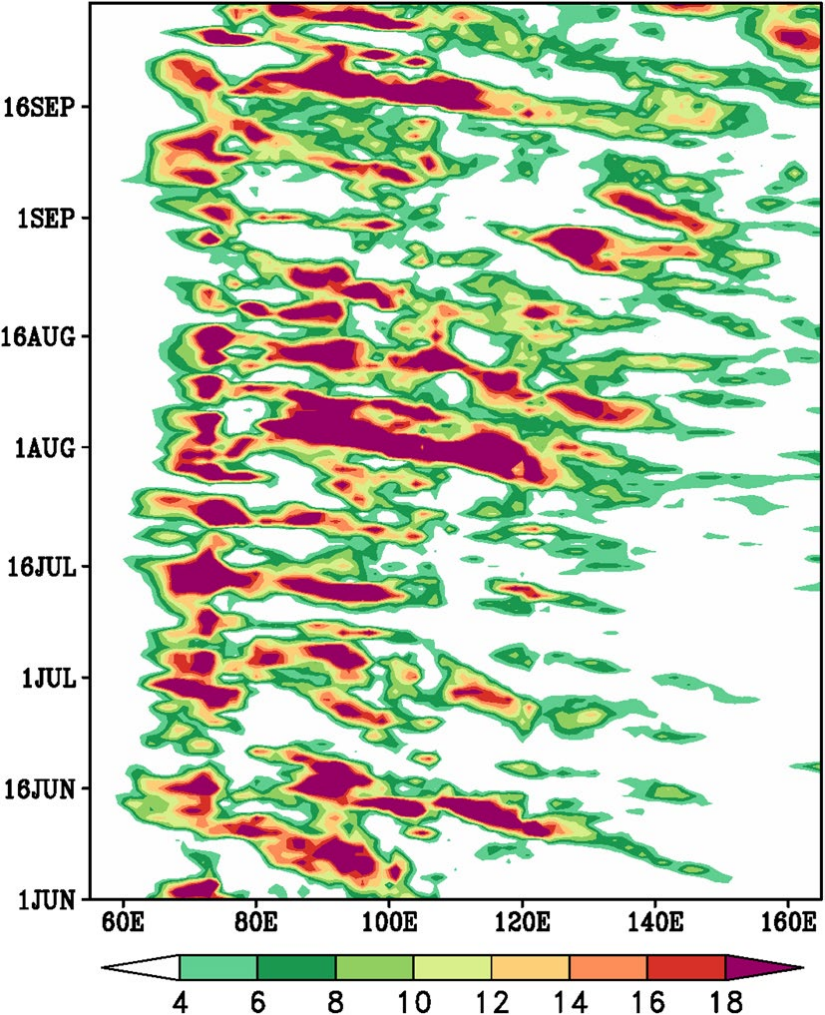
East to west propagation (time-longitude section) of rainfall (mm/day) averaged over the latitude belt (10°N–20°N), during the summer monsoon season of 2020. GPCP daily rainfall data is used to derive the rainfall propagation characteristics

## CORONA virus lockdown initiatives and its impact on air quality

The Coronavirus, namely COVID-19 was first identified in Wuhan city in the Hubei province in China in December 2019. This virus was first reported to the World Health Organization (WHO) on 31 December 2019 and became a global public health problem. The COVID-19 pandemic has been deemed a global health crisis. To control the spread of COVID-19, various administrative regulations included lockdowns were implemented throughout the world to prevent the outbreak of COVID-19 (Vuong et al. 2020). Due to the strict enforcement of vehicular movement, the emission levels reduced drastically. Such a situation has resulted in an unprecedented effect on the environment. As a result, air quality has significantly improved. The lockdown has led to a reduction of air pollutants throughout the world, including South and East Asia mainly due to the low density of vehicles (road, rail and air) circulating in cities. Thus, the reduction of air pollutants is one of the positive effects of COVID-19 lockdown on the environment (Vuong et al. 2020; Chauhan and Singh 2020; Mahato et al. 2020; Manmeet et al. 2021; Fadnavis et al. 2021).

Very strict lockdown measures were implemented by India and China due to their vast population (Metya et al. 2020). Results suggest the lockdown over China also had the effect of lowering the concentration of PM2.5 (Ma and Kang 2020). The enforced lockdown amid the COVID-19 pandemic eased anthropogenic activities across India. The satellite derived AOD and absorption AOD showed a significant reduction of ~ 30% over the Indo-Gangetic Plains in north India. All the measured pollutants showed significant reduction during the entire lockdown, improved the air quality and the environment (Srivastava et al. 2021).

Figure 7 displays the Aerosol Optical Depth (AOD) data during the onset phase of the southwest monsoon (May–June). Vast regions of the AOD layers are visible from the Arabian Peninsula up to the Indian subcontinent (Fig. 7a) based on the 20-year climatology (1996–2015) over the South Asian monsoon region. Likewise, AOD layers are also visible over the East Asian monsoon region over China and the Korea-Japan sector. A similar pattern is discernable during the onset of the monsoon 2020 (May–June 2020), however, with much less intensity, implying the reduction in AOD layers following the commencement of the lockdown initiatives. This reduction in AOD layers is clearly brought out by examining the difference between the top two panels (Fig. 7b, a). Significant reduction in AOD layers by about 20% over the Arabian Sea, central parts of India, the Bay of Bengal and over the regions of the Yangzte River Valley, the Korea-Japan peninsulas can be inferred (Fig. 7c).

**Fig. 7:**
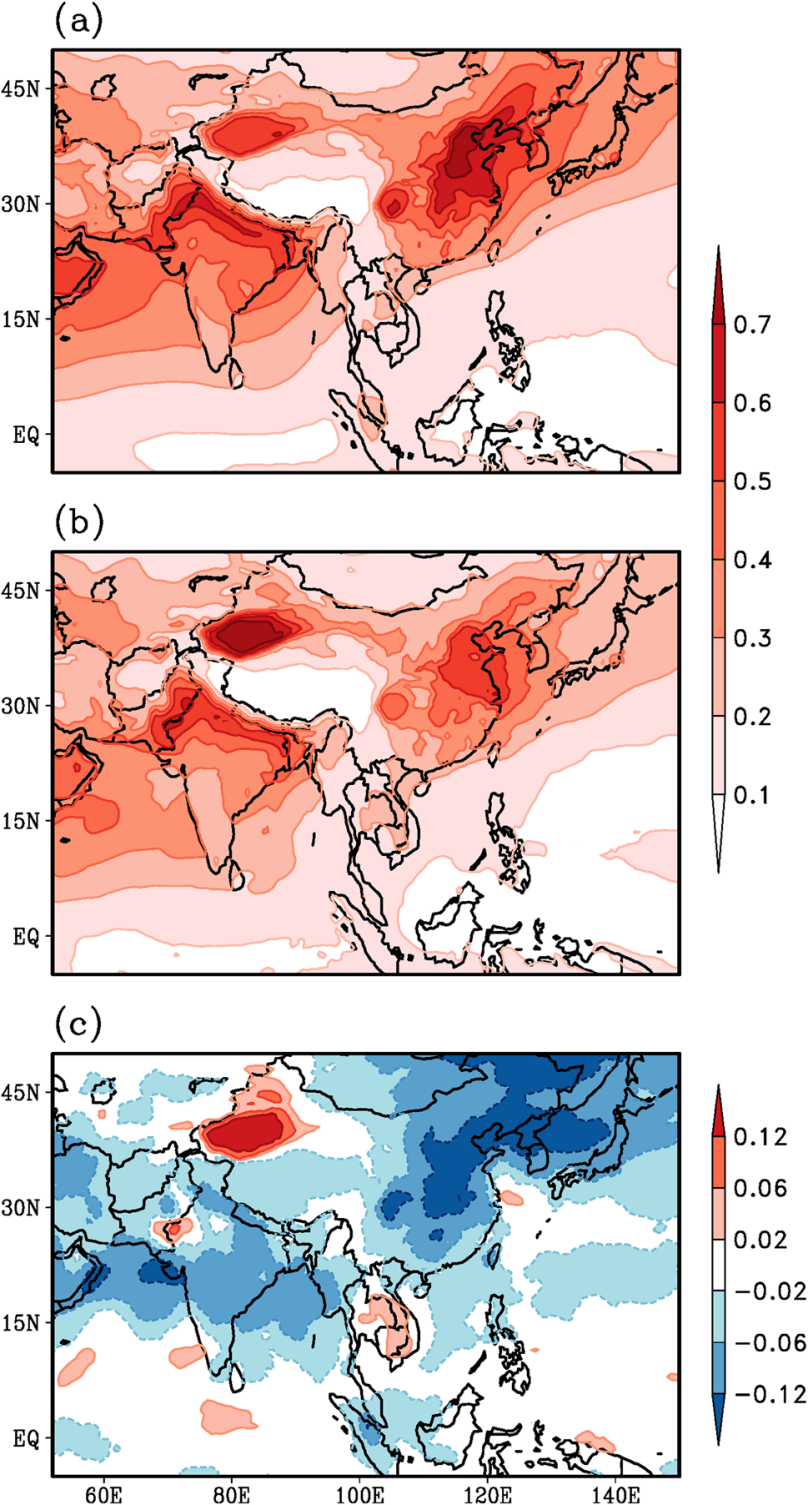
Spatial variation of May–June AOD as derived from MEERA2 reanalysis product. **a** Climatology for the period 1996–2015, **b** Monsoon 2020 and **c** anomaly for monsoon 2020, obtained by subtracting (**a**) from (**b**)

## Role of aerosols

As indicated earlier, besides the dominant roles of the ITCZ and the ocean-land thermal contrast over the South and East Asian monsoons, aerosols from both natural and anthropogenic emissions can further modulate these two monsoon systems. The heavy aerosol layers can cool the surface by scattering and absorbing the solar radiation, which reduces the ocean-land thermal contrast and weakens the monsoons. On the other hand, absorbing aerosols can heat the mid-troposphere pumping the air upwards and strengthening the monsoon. Thus, absorbing aerosols can modify the circulation and precipitation over both the South and the East Asian monsoon regions (Jin et al. 2021). The Arabian Peninsula is a key source of desert dust affecting the South Asian Summer Monsoon. A major source of dust over East Asia originates from the Mongolian Gobi Desert and the Taklimakan desert. The induced changes in circulation distribution due to aerosols can also result in enhanced (reduced) precipitation over the Indian sub-continent (South China) (Wang et al. 2021a, b).

A number of studies have shown that aerosols have substantial effects on precipitation and precipitation extremes (Ramanathan et al. 2005; Polson et al. 2014; Sanap and Pandithurai 2015; Sanap et al. 2015) via complex and in some cases, competing mechanisms. For instance, increases in aerosols during the twentieth century have been found to contribute remarkably to the decrease in Northern Hemisphere precipitation (Polson et al. 2013), the meridional shift of the ITCZ (Hwang et al. 2013), the weakening trend of the South Asian monsoon (Bollasino et al. 2011, 2014; Chung and Ramanathan 2006), the so-called southern flood-northern drought pattern over East China (Gong and Ho 2002) as well as a shift of rainfall towards heavy mode over East China (Ma et al. 2017). Rahul et al. (2008) showed that high (low) aerosol loadings over the Arabian Sea are closely related to the enhanced (reduced) Indian monsoon rainfall in July. The aerosol reduction could result in an increased likelihood of extreme precipitation and related disasters. This is particularly important over East Asia in accordance with the larger magnitude of aerosol reduction compared to South Asia (Zhao et al. 2019). More recently, it was suggested that future aerosols reduction would lead to a significant increase in precipitation extremes over Asia (Wang et al. 2016). Another recent study documents a decreasing rainfall over the monsoon regions due to aerosol forcing (Ha et al 2020). Thus, the extent to which anthropogenic aerosols weaken or strengthen the monsoons and underlying mechanism is still largely uncertain (Jin et al. 2021).

## AOD reduction and monsoon 2020 variability

The reduction in the AOD layers (Fig. 7c), the strong shortwave radiation without the dimming effect of aerosols eventually lead to early heating of land as well as ocean and result in a strong thermal contrast between land and ocean. Furthermore, we expect the maximum surface heating and the thermal contrast to set up before the commencement of the monsoon in June. The anomalous surface temperature pattern during the month of May (Fig. 8c) depicts much higher warming over East Asia, in particular from the Indochina peninsula to eastern China, than over South Asia. Even the meridional thermal contrast between the land and the ocean appears to be much stronger over the East Asian sector (100–130°E, Fig. 8c) compared to that of South Asia (60–90°E). However, the AOD anomaly pattern (Fig. 7c) reveals a reduction from eastern China to northeast Asia and from the Arabian Peninsula to the Indian subcontinent. A comparison of Figs. 1c, 7c and 8c reveals two distinct regions, West-Central India and Yangzte River Valley, where rainfall enhancement (Fig. 1c), AOD reduction (Fig. 7c) and surface temperature warming (Fig. 8c) match very well (blocks 3 and 5 in Fig. 9). Thus, the reduced aerosols (Fig. 7c) during the onset phase of summer monsoon 2020 have resulted in higher surface temperatures and a stronger thermal contrast between land and ocean (Fig. 8c), a stronger monsoon circulation (Fig. 3c), stronger moisture transport and convergence from the oceans towards the land-masses (Fig. 5c) resulting in enhanced rainfall (Fig. 1c), particularly over West-Central India and the Yangzte River valley.

**Fig. 8:**
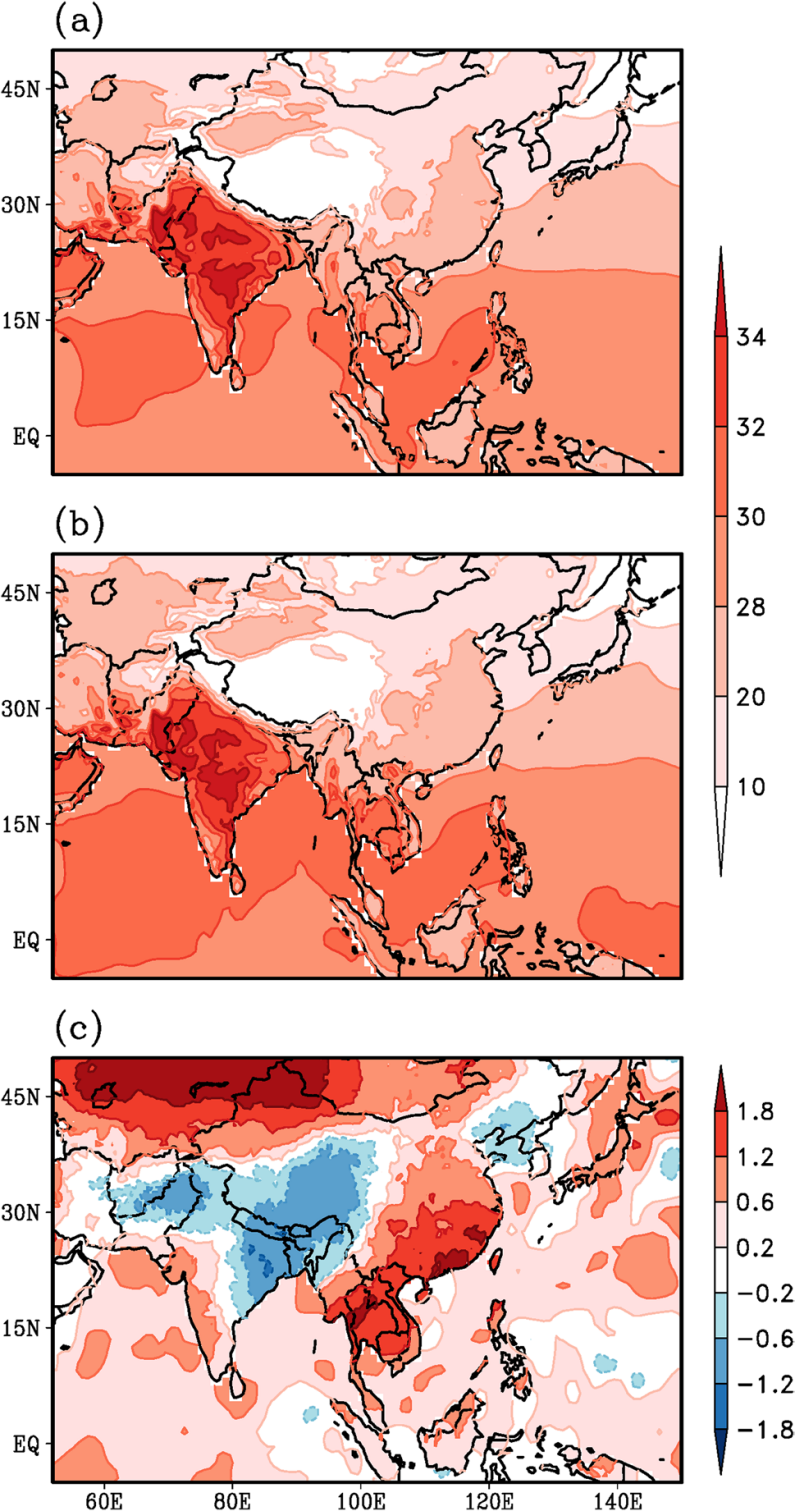
Spatial variation of surface temperature (°C) during the month of May, as derived from CRU (over land) and HadISST (over ocean) datasets. **a** Climatology for the period 1996–2015, **b** monsoon 2020 and **c** anomaly for monsoon 2020, obtained by subtracting (**a**) from (**b**)

**Fig. 9:**
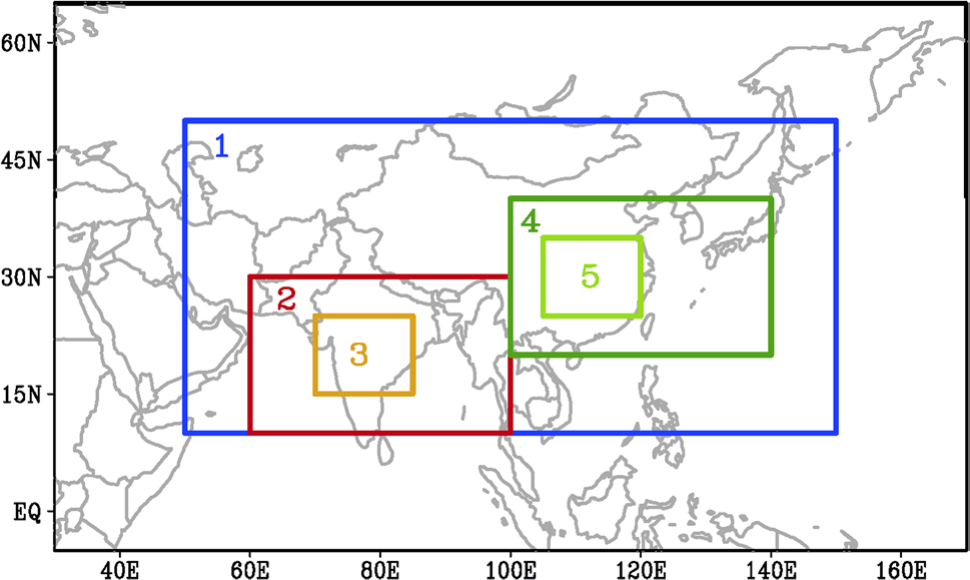
Regions selected to perform pattern correlation analysis between anomalous summer monsoon rainfall (Fig. 1c) and May–June AOD (Fig. 7c). Region 1. Asian domain (10–50°N, 50–150°E), 2. South Asia (10–30°N, 60–100°E), 3. West-Central India (15–25°N, 70–85°E), 4. East Asia (20–40°N, 100–140°E) and 5. Yangzte River Valley (25–35°N, 105–120°E)

The impact of preceding reduced aerosols on the subsequent monsoon rainfall is quantified by computing the Anomaly Correlation Coefficient (ACC) between these two patterns (Figs. 1c and 7c). The ACC is one of the most widely used measure in the verification of spatial fields and to determine the similarity between patterns. The significance of the ACC is normally quantified in percentage by the expression (ACC)^2^ × 100 (von Storch and Zwiers 1999). The anomaly patterns for monsoon 2020 are computed by subtracting the 20-year mean from the monsoon 2020 pattern. The pattern correlation coefficient between AOD and rainfall have been performed after re-gridding the AOD data, having a horizontal resolution of 0.625 × 0.5°, to the resolution of the CRU rainfall data (0.5 × 0.5°) using bilinear interpolation technique.

These computations are done for 5 regions (Fig. 9): the Asian domain (block 1 in Fig. 9), the South (block 2) and East Asian (block 4) domains, West-Central India (block 3) and the Yangzte River Valley (block 5). Our main focus will be on blocks 3 and 5. Number of grids implies the number of values on which the anomaly correlations are computed (Table 1). A cursory glance at the results (Table 1) reveals the magnitudes of the relationship are stronger over East Asia than over South Asia. Over South Asia the West-Central India region (Block 3) displays the maximum strength (ACC = − 0.58) and over East Asia the Yangzte River Valley displays the maximum strength (ACC = − 0.82). An ACC = − 0.82 would imply that about 70% {(− 0.82)^2^ × 100} of the unprecedented heavy summer monsoon rainfall variability over the Yangzte River Valley region could be associated with the preceding May–June reduced AOD, whereas only 35% {(− 0.58)^2^ × 100} of rainfall variability over West-Central India could be associated with the preceding AOD, implying that the reduced aerosols due to lockdown initiatives may have played a role in the erratic Asian Monsoon 2020. However, the ACC value of − 0.82 over the Yangzte River Valley reduces to − 0.58 on removing the possible impact of surface temperature (partial correlation analysis). Furthermore, the ACC value of 0.72 between rainfall and surface temperature over Yangzte River Valley reduces to near zero (0.02) on removing the impact of AOD. Similar inferences can be drawn over the East Asian region (region 4). This would imply that the rainfall over East Asia in particular the Yangzte River Valley is more associated with the preceding aerosols than the preceding surface temperature heating. Over West-Central India the ACC of − 0.58 (0.37) between rainfall and AOD (surface temperature) remains the same even after the effect of surface temperature (AOD) is removed, implying that both are contributing to the rainfall variability, about 35% by AOD and 15% {(0.37)^2^ × 100} by surface temperature heating. Surface temperature appears to have no impact on rainfall over the entire Asian domain (region 1) and the South Asian domain (region 2) as the ACCs are near zero (Table 1 column 3). Thus, the preceding reduced aerosol layers could be one of the causes for the subsequent enhancement of summer monsoon rainfall over the Yangzte River Valley and West-Central India. The analysis further reveal that preceding surface temperature may also have some role over West-Central India but not over Yangzte River Valley.

**Table 1:** Pattern correlation coefficient of anomalous (climatological mean removed) rainfall pattern (Fig. 1c) with AOD (Fig. 7c) and surface temperature (Fig. 8c) patterns, for land points over 5 different regions over the Asian domain (see Fig. 9)

		ACC: Rainfall and AOD	ACC: Rainfall and Surface temperature	No. of grids
1	Asian domain (10–50N, 50–150E)	− 0.41 (− 0.40)	0.09 (0.01)	10,103
2	South Asia (10–30N, 60–100E)	− 0.34 (− 0.34)	0.07 (0.09)	1865
3	West-Central India (15–25N, 70–85E)	− 0.58 (− 0.58)	0.37 (0.38)	502
4	East Asia (20–40N, 100–140E)	− 0.57 (− 0.54)	0.27 (− 0.18)	1851
5	Yangzte River Valley (25–35N, 105–120E)	− 0.82 (− 0.58)	0.72 (0.02)	599

*ACC* Anomaly Correlation Coefficient

Pattern correlation coefficient of rainfall and AOD (surface temperature) after removing the effect of surface temperature (AOD) are given in brackets

Number of grids i.e. no of values used to compute the correlation coefficients

## Discussion and summary

This study examined the possible drivers for the highly unprecedented heavy amounts of rains over South Asia and East Asia during monsoon season (June through September) 2020 resulting in catastrophic floods and severe damages. The simultaneous heavy rains over South and East Asia have been a very rare event in the past history of these monsoon sub-systems. Several factors may have contributed to the unprecedented heavy rains over South and East Asia as documented by various recent publications. The summer monsoon 2020 period was marked by excessive lockdown measures implemented by government agencies to control the spread of the COVID-19 virus. These lockdown initiatives resulted in a considerable reduction in air pollutants/aerosols and impact on the environment due to the practical standstill of the travel industry—rail, road and air. This has been the main motivation of this study to determine whether the reduced pollutants/aerosols had any role in the atmospheric circulation and the unprecedented heavy rains.

Examination of the climate characteristics—lower tropospheric atmospheric circulation, sea surface temperatures over the Indian and the Pacific Oceans, moisture flux transport and convergence during summer monsoon 2020 synchronized very well with the heavy rains over the broad Asian domain of South and East Asia. The striking feature of the atmospheric circulation was the considerable westward shift of the NPSH into the South China Sea. The easterly trade wind anomalies over the southern edge of the NPSH penetrated over the Indian region. In conjunction with this anomalous wind flow across the Indian region supported the generation of strong anomalous cyclonic circulation over the Arabian Sea adjacent to the West-Coast of India, resulting in heavy rains over the West-Central India and peninsular India. One of the factors leading to heavy rainfall events over East Asia is the low-level southerly jet at the western edge of the NPSH inducing moisture flux convergence into the quasi-stationary Meiyu-Changma-Baiu front (Guan et al. 2020; Wolf et al. 2021).

The incoming solar radiation intensified the thermal contrast between land and ocean over South and East Asia due to considerable reduction in aerosols, resulting in the intensified monsoon flow and heavy rains. Similarity between the anomalous rainfall pattern and the anomalous AOD pattern, with a high negative relation, was more discernable over West-Central India and the Yangzte River Valley. Results further reveal that nearly 70% of the heavy rains over the Yangzte River Valley could be associated with the preceding May–June reduced aerosols, while the heavy rains over West-Central India could be associated with reduced aerosols (about 35%) as well as surface temperature warming (about 15%) during the month of May. A schematic flow chart illustrating the impact of lockdown regulations on the environment and the subsequent monsoon system is shown in Fig. 10.

**Fig. 10:**
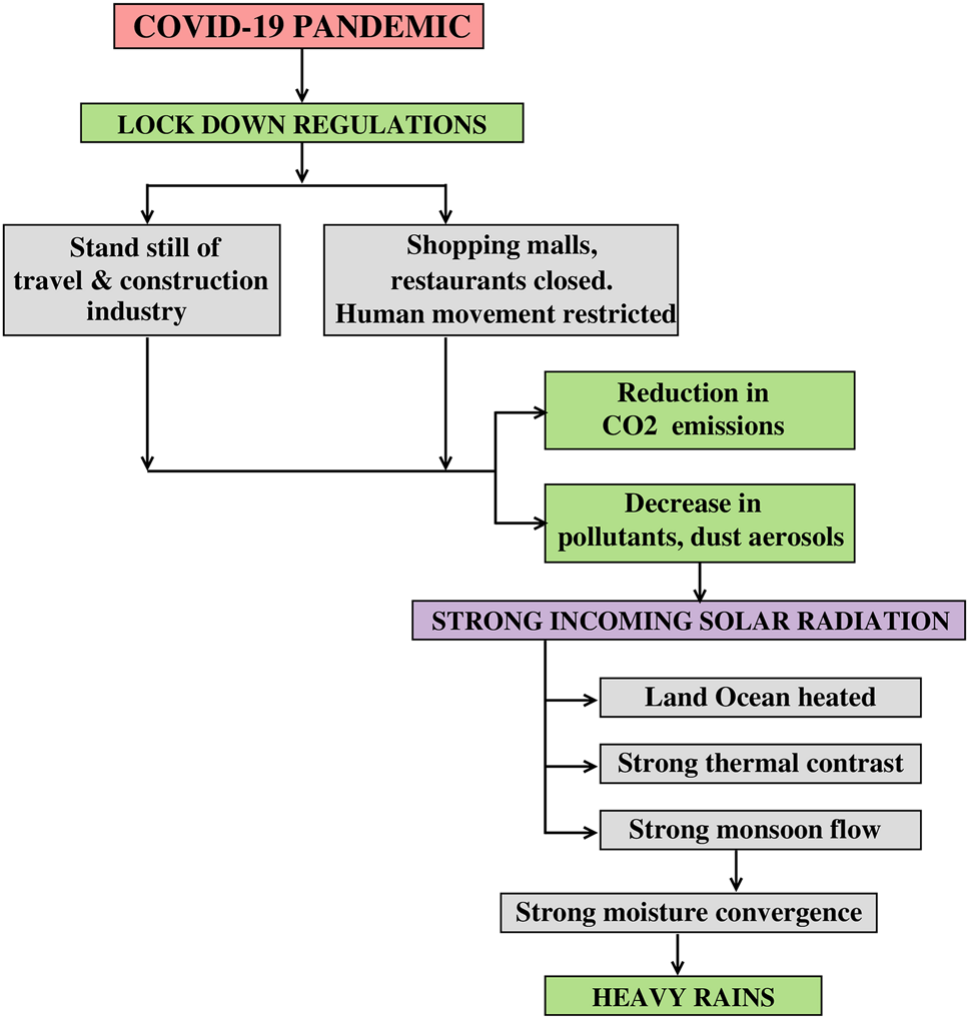
Schematic flow chart illustrating the impact of lockdown regulations on the environment and the subsequent monsoon system

The erratic nature of the monsoon remains a cause for concern. Can we envisage such events to repeat in the future? In spite of the uncertainty, studies have documented a robust increase in summer precipitation and intensification of the inter-annual variability of the Asian summer monsoon (Kamizawa and Takahashi 2018). An increase in monsoon rainfall over the land areas of South and East Asia is also projected, with high confidence (Wang et al. 2021a, b). A recent study foresees much stronger Indian monsoon, more erratic under the catastrophic climate change (Katzenberger et al. 2021). Time will tell whether such erratic events will occur in the future.

## Data Availability

All the data used in the present study are freely available. Details are included in the article.
